# Kinetic, Structural, and Mutational Analysis of Acyl-CoA Carboxylase From *Thermobifida fusca* YX

**DOI:** 10.3389/fmolb.2020.615614

**Published:** 2021-01-12

**Authors:** Kiran-Kumar Shivaiah, Bryon Upton, Basil J. Nikolau

**Affiliations:** ^1^Roy J. Carver Department of Biochemistry, Biophysics, and Molecular Biology, Iowa State University, Ames, IA, United States; ^2^Center for Biorenewable Chemicals (CBiRC), Iowa State University, Ames, IA, United States; ^3^Center for Metabolic Biology, Iowa State University, Ames, IA, United States

**Keywords:** acyl-CoA, biotin-dependent carboxylases, *Thermobifida fusca* YX, site-directed mutagenesis, enzyme kinetics

## Abstract

Acyl-CoA carboxylases (AcCCase) are biotin-dependent enzymes that are capable of carboxylating more than one short chain acyl-CoA substrate. We have conducted structural and kinetic analyses of such an AcCCase from *Thermobifida fusca* YX, which exhibits promiscuity in carboxylating acetyl-CoA, propionyl-CoA, and butyryl-CoA. The enzyme consists of two catalytic subunits (TfAcCCA and TfAcCCB) and a non-catalytic subunit, TfAcCCE, and is organized in quaternary structure with a A_6_B_6_E_6_ stoichiometry. Moreover, this holoenzyme structure appears to be primarily assembled from two A_3_ and a B_6_E_6_ subcomplexes. The role of the TfAcCCE subunit is to facilitate the assembly of the holoenzyme complex, and thereby activate catalysis. Based on prior studies of an AcCCase from *Streptomyces coelicolor*, we explored whether a conserved Asp residue in the TfAcCCB subunit may have a role in determining the substrate selectivity of these types of enzymes. Mutating this D427 residue resulted in alterations in the substrate specificity of the TfAcCCase, increasing proficiency for carboxylating acetyl-CoA, while decreasing carboxylation proficiency with propionyl-CoA and butyryl-CoA. Collectively these results suggest that residue D427 of AcCCB subunits is an important, but not sole determinant of the substrate specificity of AcCCase enzymes.

## Introduction

**Scheme 1:**
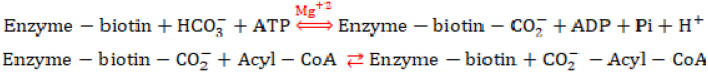
Reaction mechanism of biotin-dependent carboxylase enzymes.

Acyl-CoA carboxylase (AcCCase) is a biotin-dependent enzyme capable of carboxylating several short-chain acyl-CoA substrates (Gago et al., 2011). Typical of biotin-dependent enzymes, AcCCase catalysis involves two half reactions (Scheme 1): (a) the ATPase-coupled carboxylation of the biotin prosthetic group, catalyzed by the biotin carboxylase (BC) subunit; and (b) the transfer of the carboxyl group from the biotin-prosthetic group to the final acyl-CoA substrate, catalyzed by the carboxyltransferase (CT) subunit.

Subsequently, the “activated,” carboxylated acyl-CoA product can be utilized as a substrate in a Claisen condensation reaction catalyzed by such enzymes as fatty acid synthase (FAS) or polyketide synthase (PKS) (Beld et al., 2015; Risdian et al., 2019). For example, AcCCase from Actinobacteria are well studied because they provide building blocks for the biosynthesis of polyketides (Pfeifer et al., 2001), and depending on whether they carboxylate propionyl-CoA (generating methylmalonyl-CoA) or butyryl-CoA (generating ethylmalonyl-CoA), the subsequent PKS-catalyzed reaction will generate polyketides containing either methyl or ethyl branches, respectively (Cane et al., 1998; Khosla and Keasling, 2003). This enzymatic flexibility ensures that these organisms have the metabolic flexibility to produce a wide range of natural products, which have potential applications as antibiotics, anticancer agents, cholesterol lowering drugs, and other pharmacological reagents (Hopwood and Sherman, 1990; Cane et al., 1998).

In a broader context, biotin-dependent enzymes catalyze carboxylation, decarboxylation or transcarboxylation reactions (Cronan and Waldrop, 2002; Nikolau et al., 2003; Tong, 2013). The majority of biotin-dependent carboxylases catalyze the carboxylation of acyl-CoA esters of organic acids and are aptly identified by the specific acyl-CoA substrate (i.e., acetyl-CoA carboxylase, propionyl-CoA carboxylase, 3-methylcrotonyl-CoA carboxylase) (Waldrop et al., 2012; Tong, 2017). Other substrates that are carboxylated by such enzymes include pyruvate or urea (St. Maurice et al., 2007; Xiang and Tong, 2008; Fan et al., 2012). Collectively, each of these enzymes display a relatively narrow substrate specificity, with a high preference for the one specified substrate. In contrast, the AcCCase enzyme that was the focus of the current study, displays a broader substrate specificity, being capable of carboxylating, with near equal efficiency, multiple acyl-CoA substrates; namely acetyl-CoA, propionyl-CoA and butyryl-CoA. Biotin-dependent enzymes have also been categorized based on their tertiary and quaternary organizations (Tong, 2013). According to this classification system, AcCCases belonging to a family of enzymes that are composed of three subunits: (a) the A-subunit that carries the BC and the biotin-carrier functionality (often referred to as BCCP); (b) the B-subunit that carries the CT functionality; and (c) a small, non-catalytic E-subunit (Gago et al., 2011). This type of quaternary organization is shared by two highly homologous multisubunit enzymes from *Streptomyces coelicolor*, but they display distinct substrate specificities. These enzymes are propionyl-CoA carboxylase (ScPCCase), which is highly specific for carboxylating propionyl-CoA, and producing methylmalonyl-CoA (Diacovich et al., 2002), and an AcCCase that we have for convenience of this publication labeled as ScAcCCase, and this enzyme can carboxylate acetyl-CoA, propionyl-CoA, and butyryl-CoA (Rodríguez and Gramajo, 1999; Rodríguez et al., 2001; Diacovich et al., 2002). Both ScPCCase and ScAcCCase share the same biotinylated A-subunit (i.e., AcCCA2), but they utilize different B-subunits and E-subunits, each of which share ~71 and ~58% sequence similarities, respectively (Rodríguez and Gramajo, 1999; Diacovich et al., 2004; Gago et al., 2011).

In this study we characterized an AcCCase isolated from *Thermobifida fusc*a YX, a thermophilic Actinobacterium that is commonly found growing on compost heaps, manure piles and organic waste, where temperatures can reach 55°C. These characterizations focused on the quaternary organization of the three subunits that constitute the TfAcCCAse holoenzyme, and further evaluated a potential substrate specifying residue previously identified with the ScPCCase and ScAcCCase enzymes (Diacovich et al., 2004; Arabolaza et al., 2010). We specifically focused on characterizing the TfAcCCase as this organism has prospective biotechnological applications associated with potentially producing novel bioproducts from the biodegradation of cellulosic and lignocellulosic plant biomass (Wilson, 2004; McGrath and Wilson, 2006). As with many Actinobacteria, which are well known for producing a variety of natural products that have biotechnological applications (i.e., antibiotics, insecticides, herbicides, and antifungals) (Barka et al., 2016), *T. fusca* appears to be able to produce polyketides and other lipids, which could have similar applications (Vanee et al., 2017).

## Methods and Materials

### Recombinant Expression Vectors

ORFs encoding the TfAcCCB (Tfu_2555; WP_011292978), TfAcCCA (Tfu_2557; WP_011292980), and TfAcCCE (Tfu_2556; WP_011292979) proteins were initially identified using The Thioester-active enzYme database (ThYme) (http://www.enzyme.cbirc.iastate.edu/) (Cantu et al., 2010, 2011; Chen et al., 2011). The respective ORFs were codon optimized for expression in *E. coli* and chemically synthesized as “gBlocks” (Integrated DNA Technologies, Coralville, IA, USA). The assembled ORFs were PCR amplified, using suitable primers (Supplementary Table 1), and the resulting assembled fragments were used to construct expression vectors. These vectors included pCDFDuet-1 (MilliporeSigma, MA, USA), which was used for the co-expression of N-terminal 6X-His tagged TfAcCCB (cloned into MCSI at the restriction enzyme sites, *Bam*HI and *Afl*II), with a C-terminal S-tagged TfAcCCE (cloned into MCSII at the *Nde*I and *Xho*I restriction enzyme sites) (Raines et al., 2000). The TfAcCCA ORF was cloned into pET30F vector, at the restriction enzymes sites, *Bam*HI and *Xho*I. TfAcCCA and TfAcCCE were individually expressed from pETDuet-1 vectors, with the ORFs cloned between the *Nco*I and *Not*I restriction sites of the MCS1; in these constructs TfAcCCA and TfAcCCE were expressed without any tag. The authenticity of all vector constructs was determined by DNA sequencing, conducted at Iowa State University's DNA Facility.

### Protein Expression, Purification, and SDS-PAGE Analysis

Expression plasmid constructs were harbored in *E. coli* strain BL21 λ(DE3), which were selected by the ability of the strain to grow in LB medium supplemented with the appropriate antibiotic: streptomycin (50 μg/ml) for pCDFDuet-1-based vectors, ampicillin (50 μg/ml) and kanamycin (30 μg/ml) for pET30F-based vectors. Overnight liquid pre-cultures were diluted 1:100 into fresh media (500 ml), and growth was continued at 37°C with agitation at a rate of 250 revolutions/min. Protein expression was induced by adding 0.4 mM IPTG when the A600 of the culture reached ~0.6–0.8. Following another 8 h of growth, cells were harvested by centrifugation, and cell pellets were flash frozen in liquid N_2_ and stored at −80°C until further use. Additional experiments were conducted to ensure that biotinylation was not limiting the recovery of active AcCCase enzyme. Specifically, cultures of strains expressing TfAcCCase components were supplemented with 1 mg/ml biotin. Comparative analysis of recovered enzyme from cultures grown with and without biotin indicted that biotinylation was not limiting the recovery of active AcCCA or AcCCase enzyme.

Cell pellets were suspended in a buffer consisting of 50 mM Tris-Cl, pH 8, 300 mM NaCl, 0.75 mM DTT, 1 mM EDTA, 10% glycerol and the recommended amount of Pierce EDTA-free protease inhibitor (Thermo Scientific, Waltham, MA). The cell suspension was sonicated on ice with 7–10 bursts, each of 15 s duration, followed by a minute of cooling. The sonicated lysate was clarified by centrifugation at 20,000 g for 30 min. The supernatant was filtered with a 0.45 μ filter (Corning Lifesciences, Corning, NY, USA) and applied to a 5 ml cOmplete™ His-tag purification Ni-NTA column (Roche Diagnostics Gmbh, Mannheim, Germany), which was equilibrated with Column Buffer (50 mM Tris-Cl, pH 8, 300 mM NaCl, 0.75 mM DTT, 1 mM EDTA, and 10% glycerol) containing 20 mM imidazole. Subsequently, the column was sequentially washed with Column Buffer containing 40 and 60 mM imidazole, and finally the His-tagged protein(s) were eluted with 10 bed volumes of Column Buffer containing 150 mM imidazole. Eluted protein-fractions were dialyzed overnight against 100 mM potassium phosphate buffer, pH 7.6, containing 0.75 mM DTT, 1 mM EDTA and 20% glycerol, and finally concentrated using an Amicon® Ultra-15 centrifugal filters (MilliporeSigma, Billerica, MA, USA). Protein samples were flash frozen in liquid N_2_ and stored at −80°C. Purified protein fractions were analyzed by SDS-PAGE (Laemmli, 1970), and protein bands were visualized by staining the gels with Coomassie Brilliant Blue R-250. The relative intensity of the stained protein bands was quantified with ChemiDoc XRS^+^ using the software Image Lab 6.0 (Bio-Rad, Hercules, CA).

### Site-Directed Mutagenesis

The D427I variant of *TfAcCCB* ORF was carried out using the Qiagen QuickChange kit (Agilent Technologies, Santa Clara, CA, USA), using the primer sequence listed in Supplementary Table 1. The mutation was confirmed by DNA sequencing (DNA Facility, Iowa State University).

### Enzyme Assays

The enzyme catalyzed carboxylation reaction was assayed via the coupled hydrolysis of ATP. Specifically, the rate of ADP formation was determined with the combination of pyruvate kinase and lactate dehydrogenase in the presence of excess phosphoenol pyruvate, resulting in the reduction in A340 due to the conversion of NADH to NAD^+^ (Grassl, 1974). Each reaction contained 100 mM potassium phosphate, pH 7.6, 5 mM MgCl_2_, 3 mM ATP, 1 mM NADH, 0.3 mg/ml BSA, 50 mM NaHCO_3_, 0.5 mM PEP, ~5 Units of pyruvate kinase and ~7 Units of lactate dehydrogenase. Reaction mixture was incubated at 37°C for 10 min, and the enzyme catalyzed reaction was initiated by the addition of the acyl-CoA substrate (MilliporeSigma, MA, USA). Enzyme catalysis was monitored continuously by reading absorbance at 340 nm using BioTek ELx808™ Absorbance Microplate Reader (BioTek US, Winooski, VT, USA).

### Size-Exclusion Chromatography

The molecular weight of purified protein complexes was determined by FPLC using a Superdex 200 10/300 GL gel filtration column (~24 ml bed volume) (GE Healthcare Life Sciences, Pittsburg, PA) using an ÄKTA FPLC system (GE Healthcare Bio-Sciences). The column was equilibrated with 100 mM potassium phosphate buffer, pH 7.6, 0.75 mM DTT, 1 mM EDTA and 20% glycerol. The column was calibrated using BioRad gel filtration molecular weight standards, which contained: bovine thyroglobulin (670 kDa), bovine γ-globulin (158 kDa), chicken ovalbumin (44 kDa), horse myoglobin (17 kDa), and Vitamin B12 (1.45 kDa). The elution volumes of these standards were compared to those of different AcCCase preparations to determine the molecular weight of the latter.

## Results

### Gene Identification

The ThYme database (Cantu et al., 2010, 2011; Chen et al., 2011) was queried with *S. coelicolor* AcCCase sequences (WP_003973461.1; ScAcCCB, AAD28554.1; ScAcCCA and WP_011030299.1; ScAcCCE) to identify genes coding for AcCCase from a thermophilic bacterium. Thereby, we identified a 3 ORF-containing operon from *T. fusca* YX, which appears to encode for subunits of a biotin-containing enzyme. The potential catalytic functionalities of the *T. fusca* subunits were computationally identified by the sequence alignments. These alignments compared the *T. fusca* sequences to homologous proteins whose catalytic capabilities have previously been biochemically well characterized; for example enzymes from *E. coli* (Guchhait et al., 1974; Li and Cronan, 1992a; Cronan and Waldrop, 2002), Arabidopsis (Nikolau et al., 2003; Sasaki and Nagano, 2004; Salie and Thelen, 2016), human (Huang et al., 2010; Tong, 2013), *S. coelicolor* (Rodríguez and Gramajo, 1999; Diacovich et al., 2002; Gago et al., 2011) and *M. tuberculosi*s (Cole et al., 1998; Li et al., 2005; Gago et al., 2006).

Based on these sequence similarities, the three *T. fusca* ORFs where characterized as: (a) Tfu_2557 (WP_011292980) encoding the TfAcCCA subunit (62.1 kDa) that appears to carry the BC and BCCP functional components; (b) Tfu_2555 (WP_011292978) encoding the TfAcCCB subunit (57.6 kDa) that carries the CT functional component; and (c) Tfu_2556 (WP_011292979) encoding the TfAcCCE subunit (8.7 kDa), a non-catalytic structural component. More specifically, the *T. fusca* AcCCB subunit appears to encompass the two domains that are homologous to the split CT-β and CT-α subunits of the *E. coli* (Li and Cronan, 1992b; Cronan and Waldrop, 2002) and plant heteromeric ACCase (Sasaki et al., 1993; Shorrosh et al., 1996; Ke et al., 2000); the CT-β and CT-α domains occur sequentially at the N-terminal and C-terminal halves of the TfAcCCB sequence. Similarly, for TfAcCCA, the BC and BCCP components can be identified as N-terminal and C-terminal domains of this protein. A significant amount of sequence similarity can also be found between *T. fusca* AcCCE ORF and the homologous proteins of ScAcCCE (~54% similarity) and ScPccE (~61% similarity) from *S. coelicolor*.

The order of these three ORFs in *T. fusca* genome is TfAcCCB-TfAcCCE-TfAcCCA, and this parallels the order of these ORFs in *Nocardiopsis gilva* (Supplementary Figure 1) (Li et al., 2013). The genus *Nocardiopsis* is affiliated with the phylum Actinobacteria, and these species display wide ecological versatility and metabolic capabilities to generate a diverse set of bioactive secondary metabolites. *N. gilva* is a halophilic bacterium, isolated from hypersaline soils, which has the ability to produce antifungal, antibacterial, and antioxidant metabolites (Tian et al., 2013). Although the broader genetic synteny appears similar among Actinobacteria, with the three AcCC ORFs adjoining the *birA* gene that encodes the enzyme that biotinylates the AcCCA subunit, the specific context appears to be more variable among this phylum, with the additional genes located between the AcCCE and AcCCA loci of the *S. coelicolor* and *M. tuberculosis* genomes (Supplementary Figure 1).

### Co-expression and Co-purification of the TfAcCCase Holoenzyme Complex

**Figure 1:**
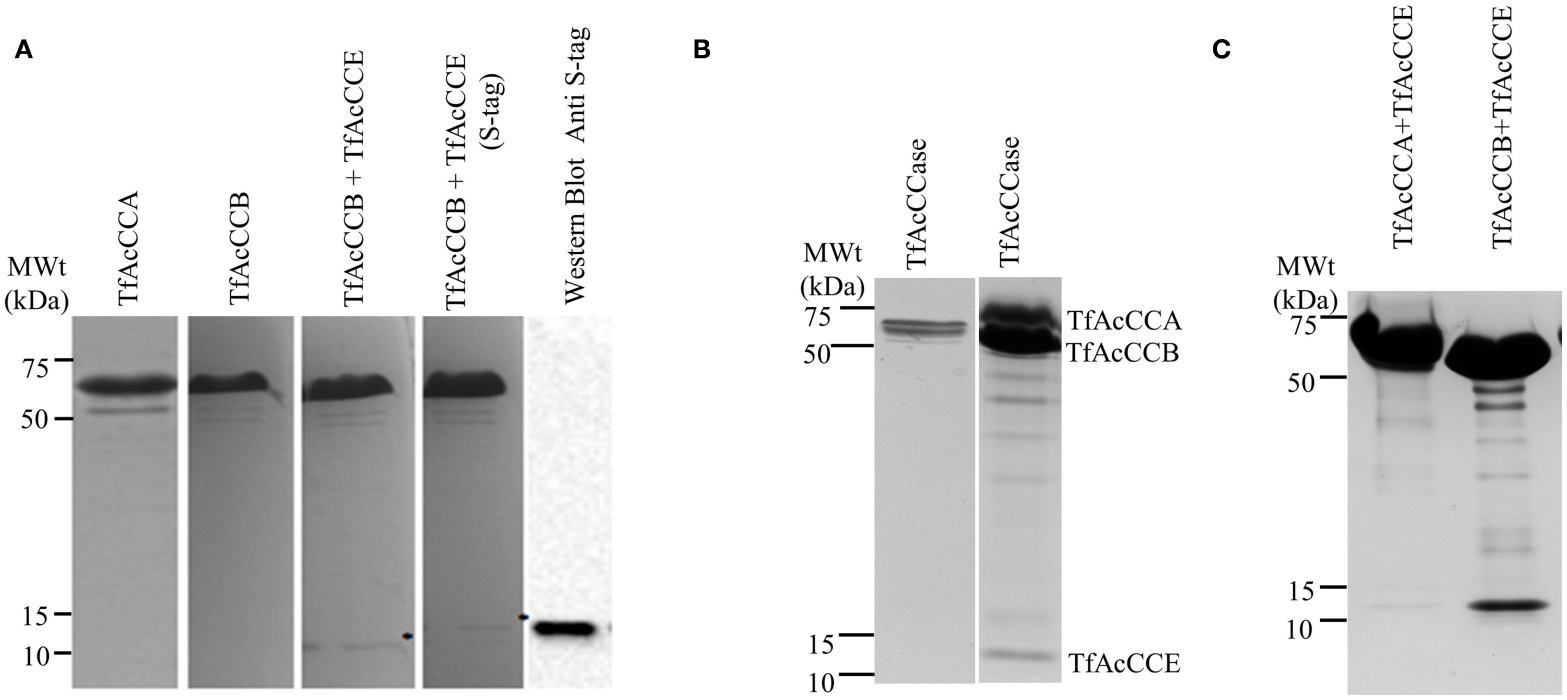
SDS-PAGE analysis of purified TfAcCCase and component subunits. **(A)** Each lane is loaded with purified preparations of the indicated purified subunits. Left to right: TfAcCCA (62.1 kDa), TfAcCCB (57.6 kDa), co-expressed TfAcCCB and TfAcCCE (8.7 kDa), and co-expressed TfAcCCB and S-tagged TfAcCCE. **(B)** Analysis of purified complex assembled by the co-expression of TfAcCCA, TfAcCCB, and TfAcCCE. Complexes were purified by affinity chromatography with a nickel affinity column via the His-tag carried by the TfAcCCB subunit. In order to visualize the presence of the TfAcCCE subunit in the purified complex, the right lane was loaded with 6 times more protein than the left lane. **(C)** Assembly of TfAcCCE into complexes with either His-tagged TfAcCCA or His-tagged TfAcCCB. Complexes were purified by affinity chromatography with a nickel affinity column via the His-tagged subunit and subjected to size-exclusion chromatography prior to SDS-PAGE analysis. Proteins were visualized by staining the resulting gels with Coomassie Brilliant Blue or visualized by Western analysis using anti-S-tag antibody.

The sequences coding for the *T. fusca* AcCCA, AcCCB, and AcCCE ORFs were recombinantly expressed in *E. coli* either individually or in different combinations in order to reconstitute and optimize the reassembly of the holoenzyme complex. In these experiments only one of the subunits was His-tagged at the N-terminus, which facilitated affinity purification with a Ni-NTA column of any complexes that formed among the co-expressed subunits. Initial expression optimization experiments revealed that the co-expression of TfAcCCE with TfAcCCB generated higher titers of the latter catalytic subunit, suggesting the stabilization of TfAcCCB by potentially being assembled into a complex with TfAcCCE. In these co-expression experiments, we separately confirmed the expression of the TfAcCCE subunit by expressing an S-tagged version of this subunit and detecting its expression by western blot analysis using the antibody against the S-tag (Figure 1A).

Following these initial expression optimization experiments, we developed an expression system in which the TfAcCCase holoenzyme was reconstituted *in vivo* by co-expressing the non-tagged TfAcCCA and TfAcCCE subunits, and simultaneously co-expressing a His-tagged TfAcCCB subunit. Figure 1B shows that affinity chromatography with a Ni-NTA column of the His-tagged TfAcCCB subunit led to the co-purification of TfAcCCA and TfAcCCE subunits, indicating that a holoenzyme complex was reconstituted and purified. Similar analyses with His-tagged TfAcCCA and His-tagged TfAcCCB, indicate that TfACCE associates with the latter subunit, but not the former (Figure 1C).

### Interactions Between Catalytic and Non-catalytic Subunits

**Figure 2:**
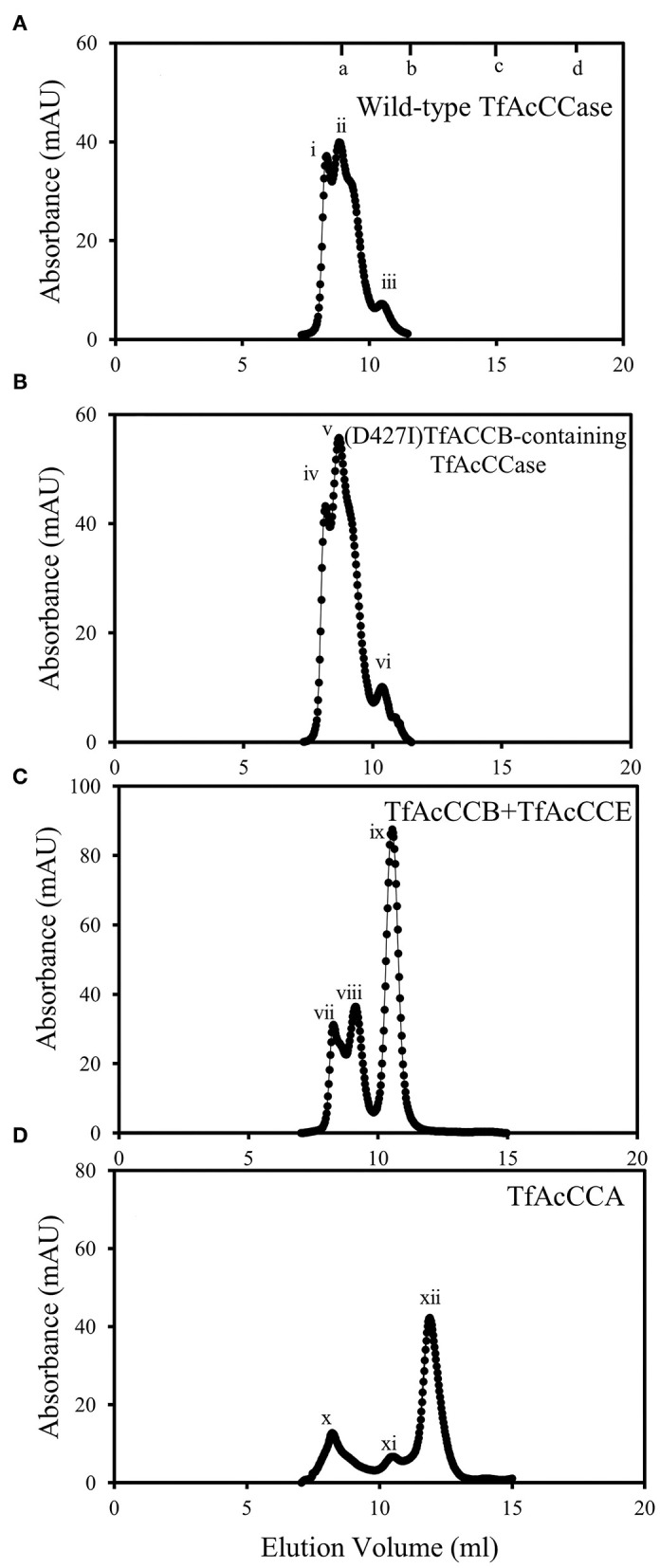
Gel filtration FPLC analysis of reassembled TfAcCCase and sub-component complexes. All reassembled complexes were purified by using a nickel affinity column via a His-tag fused to one subunit, and the purified preparations were subjected to gel-filtration chromatography. The gel filtration column was calibrated by determining the elution volume of the standard proteins: (a) bovine thyroglobulin (670 kDa); (b) bovine γ-globulin (158 kDa); (c) chicken ovalbumin (44 kDa); (d) horse myoglobin (17 kDa): **(A)** reassembled wild-type TfAcCCase; **(B)** reassembled TfAcCCase using the D427I-mutant of the TfAcCCB subunit; **(C)** His-tagged TfAcCCB-TfACCE subcomplex; **(D)** His-tagged TfAcCCA.

Gel filtration chromatography was used to determine the molecular weight of the complexes that formed when different combinations of TfAcCCE, TfAcCCB and/or TfAcCCA were co-expressed and co-purified (i.e., those analyzed by SDS-PAGE as shown in Figure 1). Co-expressing all three subunits results in the recovery of complexes that resolved as two major peaks with apparent molecular weights of 1,160 ± 45 kDa (peak i) and 880 ± 30 kDa (peak ii), and a minor peak at 350 ± 13 kDa (peak iii) (Figure 2A); all three peaks contained the entire complement of the three subunits (Supplementary Figure 2). The molecular weight of these complexes was determined at three different protein concentrations, each differing by 2-fold. These molecular weight determinations differed from each other by <5% (data not shown), indicating that each of these peaks represent stable holoenzyme complexes of different subunit stoichiometry. As demonstrated in Figure 1B, SDS-PAGE analyses indicate that in these preparations the TfAcCCA and TfAcCCB subunits occur in an equal molar ratio. Additionally, densitometric analysis of these gels indicate that the staining intensity of the TfAcCCE subunit band occurs at 6.6% (±0.5) of the intensity of both the TfAcCCA and TfAcCCB subunits. This ratio is close to the molecular weight ratio of these subunits [8.7 kDa:(62.1 kDa + 57.6 kDa) = 0.073], indicating that the 3 subunits occur at equal molar ratio. [Note, we determined the ratio of E:(A + B), rather than E:A:B, because gels had to be overloaded in order to visualize the AcCCE-subunit, which caused the merger of the AcCCA and AcCCB subunits bands, making it difficult to separately conduct densitometry of the A and B subunit]. In combination therefore, we surmise that these 3 gel-filtration elution peaks (i, ii, and iii) may be dimeric and trimeric forms of an A_3_B_3_E_3_ monomeric unit, with a preference for the formation of the dimer, (A_3_B_3_E_3_)_2_ that has a molecular weight of ~880-kD.

Analogous experiments were conducted to evaluate the role of TfAcCCE in the formation of potential subcomplexes with either TfAcCCA or TfAcCCB. The co-expression of His-tagged TfAcCCB with TfAcCCE resulted in the recovery of a subcomplex that included both subunits, whereas such a heteromeric subcomplex was not formed when His-tagged TfAcCCA was co-expressed with TfAcCCE. Based on the apparent molecular weight of the major peak recovered from the TfAcCCB-TfAcCCE subcomplex (349 ± 24 kDa; peak ix) (Figure 2C), and assuming that the two subunits occur in equimolar ratio, as occurs in the holoenzyme complex, the oligomeric state of this subcomplex would be B_6_E_6_. Less abundant peaks were also recovered in these preparations (peak vii at 1,199 ± 91 kDa and peak viii at 756.3 ± 44.7 kDa) (Figure 2C), which may therefore be higher oligomeric states, possibly (B_6_E_6_)_2_ and (B_6_E_6_)_3_, respectively. Although TfAcCCA does not appear to associate with TfAcCCE, it does form a series of subcomplexes with apparent molecular weights of 168 ± 10 kDa, 353 ± 18 kDa and 1,232 ± 69 kDa (peaks xii, xi, and x, Figure 2D); the preferred subcomplex (peak xii) maybe trimeric (A_3_) and the two minor peaks being higher multimers of this trimeric sub-complex [i.e., (A_3_)_2_ and (A_3_)_6_, respectively].

### The Role of TfAcCCE in Determining Catalytic Efficiency

**Figure 3:**
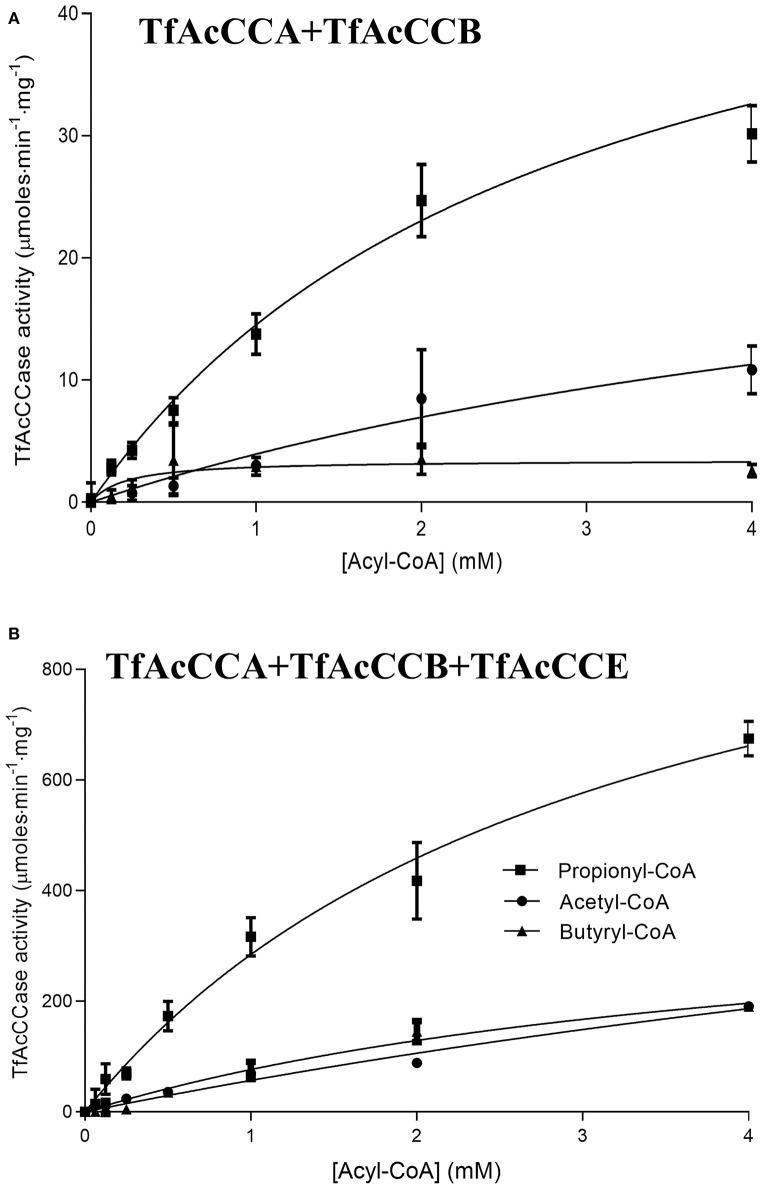
Acyl-CoA dependence of the carboxylation reaction catalyzed by TfAcCCase preparations in the absence **(A)** or presence **(B)** of the TfAcCCE subunit. Data-points are the average of triplicate assays (+SE) with either acetyl-CoA, propionyl-CoA or butyryl-CoA as the substrate. These experiments were conducted with two different enzyme preparations.

The catalytic activity of the TfAcCCase holoenzyme was assayed and compared to that of the TfAcCCE-deficient enzyme (Figures 3A,B; Table 1). These experiments were conducted by first purifying TfAcCCB from a strain that was also co-expressing TfAcCCE, or from a strain that was expressing TfAcCCB in the absence of TfAcCCE. These two preparations were mixed *in vitro* with purified TfAcCCA and assayed for catalytic activity with the three potential acyl-CoA substrates, acetyl-CoA, propionyl-CoA or butyryl-CoA. These data indicate that although the reconstituted TfAcCCE-deficient enzyme is catalytically competent, the presence of TfAcCCE subunit increases the specificity constant (*V*_max_/*K*_m_) of the reconstituted holoenzyme by about 20-fold. This enhancement of catalytic activity occurs with all three substrates that were evaluated, with propionyl-CoA being the preferred substrate, followed by acetyl-CoA and then butyryl-CoA. In all cases, the TfAcCCE-enhancement of catalytic efficiency was primarily due to increases in *V*_max_, which was increased by 20- to 100-fold depending on the substrate, with only minor changes in *K*_*m*_ values for these substrates. These kinetic parameters of the TfAcCCase enzyme are comparable to those previously determined for similar enzymes isolated from *S. coelicolor* and *M. tuberculosis* (Supplementary Table 2).

**Table 1:** The effect of inclusion of the TfAcCCE subunit on the carboxylation of propionyl-CoA, acetyl-CoA and butyryl-CoA substrates catalyzed by TfAcCCase.

**Kinetic constants**	**Acyl-CoA substrates**					
	**Propionyl-CoA**		**Acetyl-CoA**		**Butyryl-CoA**	
	**Plus TfAcCCE**	**Minus TfAcCCE**	**PlusTfAcCCE**	**Minus TfAcCCE**	**Plus TfAcCCE**	**Minus TfAcCCE**
*V*_max_(μmoles·min^−1^·mg^−1^)	*1, 186*±107	55.8 ± 6.6	771 ± 254	30 ± 15	412 ± 60	3.5 ± 0.65
*K*_m_ (mM)	3.2 ± 0.5	2.8 ± 0.6	12.7 ± 5	6.5 ± 5	4.4 ± 1	0.21 ± 0.18
*V*_max_/*K*_m_	370 ± 70	20 ± 4	61 ± 20	4.6 ± 4	94 ± 26	17 ± 16

### Role of D427 in Substrate Specificity

**Figure 4:**
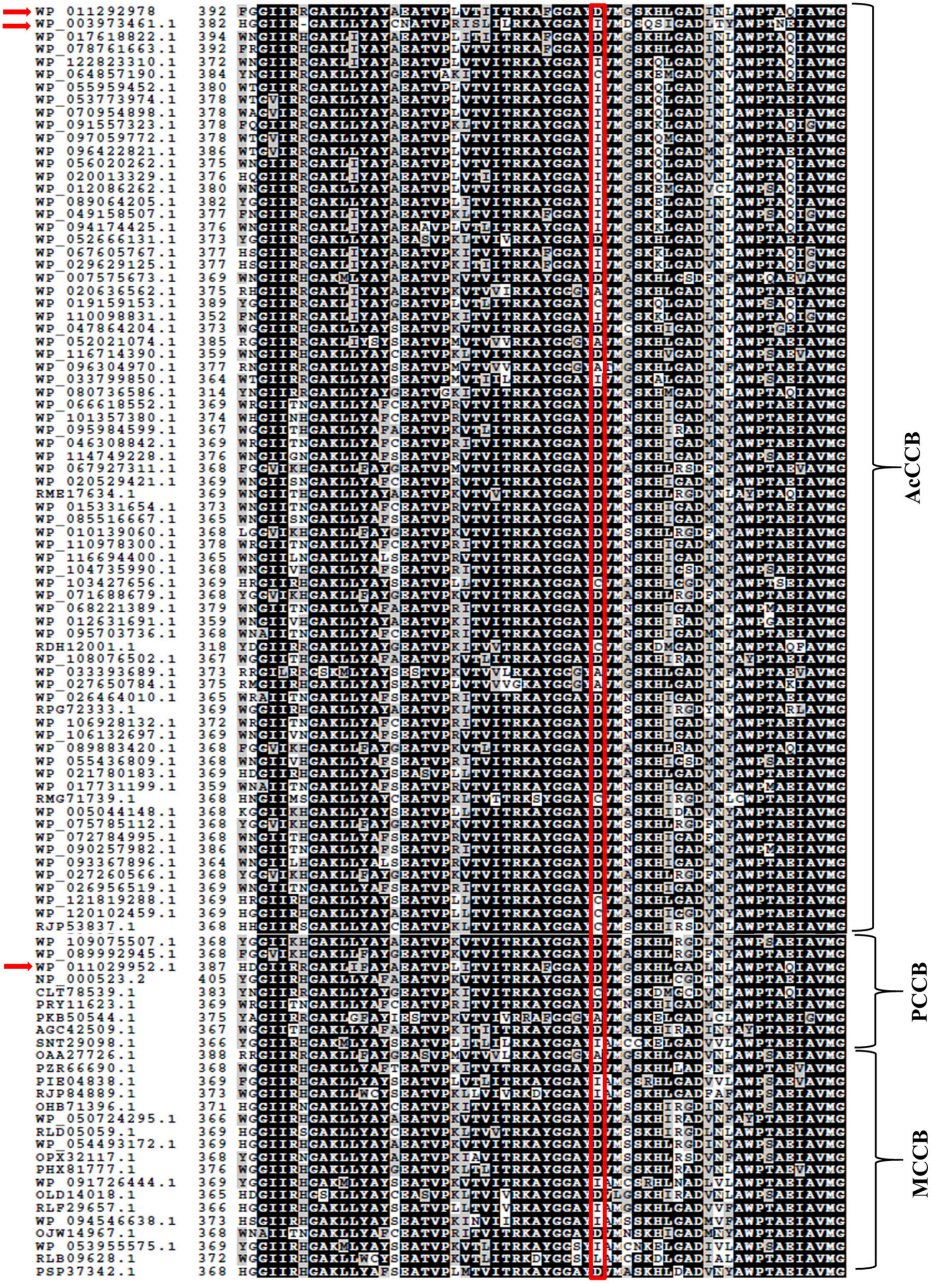
Amino acid sequence comparisons of CT subunits of AcCCase, PCCase and 3-methylcrotonyl-CoA carboxylase (MCCase) enzymes. Highlighted is the conserved Asp residue, near the C-terminus of these proteins that may play a role in determining the substrate specificity of these enzymes. The sequences of TfAcCCB (WP_011292978), ScAcCCB (WP_003973461.1), and ScPccB (WP_011029952.1) subunits are arrowed.

The selection of the organic substrate that is carboxylated by biotin-dependent enzymes is primarily a characteristic of the CT catalytic functionality, which is the 2nd half-reaction catalyzed by these enzymes. Prior characterization of the PCCase and AcCCase from *S. coelicolor* has indicated that a conserved residue (D422 of ScPccB; I420 of ScAcCCB) situated at the C-terminal end of these B subunits influences the substrate specificity of these enzymes (Diacovich et al., 2004). Comparing the amino acid sequences of TfAcCCB with ScPccB and ScAcCCB, identified that residue D427 of the former protein corresponds to residue D422 and I420 of the latter two proteins. Additional sequence comparisons establish that this residue is conserved among biotin-dependent carboxylases that can catalyze the carboxylation of propionyl-CoA, but it is less conserved in the broader context of other CT catalytic subunits that carboxylate other acyl-CoA substrates (Figure 4).

**Figure 5:**
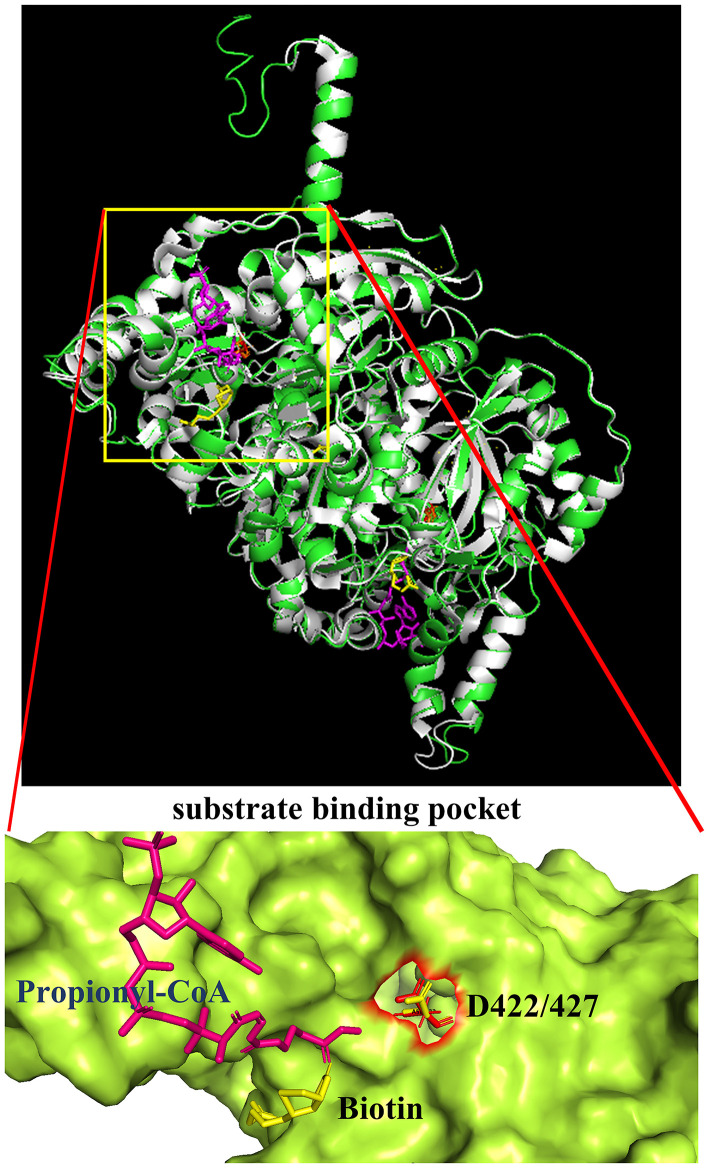
Superimposition of the TfAcCCB computational tertiary structure model developed by using I-TASSER (green) and the ScPccB experimentally determined tertiary structure (PDB: 1XNY) (gray). RMSD of the aligned atoms between the two structures is 0.3 Å (2,830 atoms aligned out of 3,797, after rejecting the 527 outlier atoms). The expanded view of the substrate binding site identifies the relative atomic positions of propionyl-CoA, biotin and D427 as determined in the PDB:1XNY structure.

Additional confirmation of the equivalence of D427 of TfAcCCB to D422 of ScPccB was gained by generating a computational model of the tertiary structure of the TfAcCCB subunit. This model was generated with I-TASSER (Roy et al., 2010; Yang et al., 2014; Yang and Zhang, 2015), which finds structural templates in PDB, using multiple threading alignment approaches. The resulting computational model was superimposed on the experimentally determined structure of *S. coelicolor* PccB (PDB: 1XNY). The two structures were aligned using PyMol (https://pymol.org/2) and they align with a root means square deviation (RMSD) of 0.3 Å (Figure 5). This tertiary structure alignment reinforced the identification of D427 of the TfAcCCB subunit as equivalent to D422 of ScPccB. Specifically, D422 of ScPccB (and D427 in the TfAcCCB model) is situated deep in the substrate binding pocket, and the side chain of this residue faces the acyl-group of the acyl-CoA substrate.

**Figure 6:**
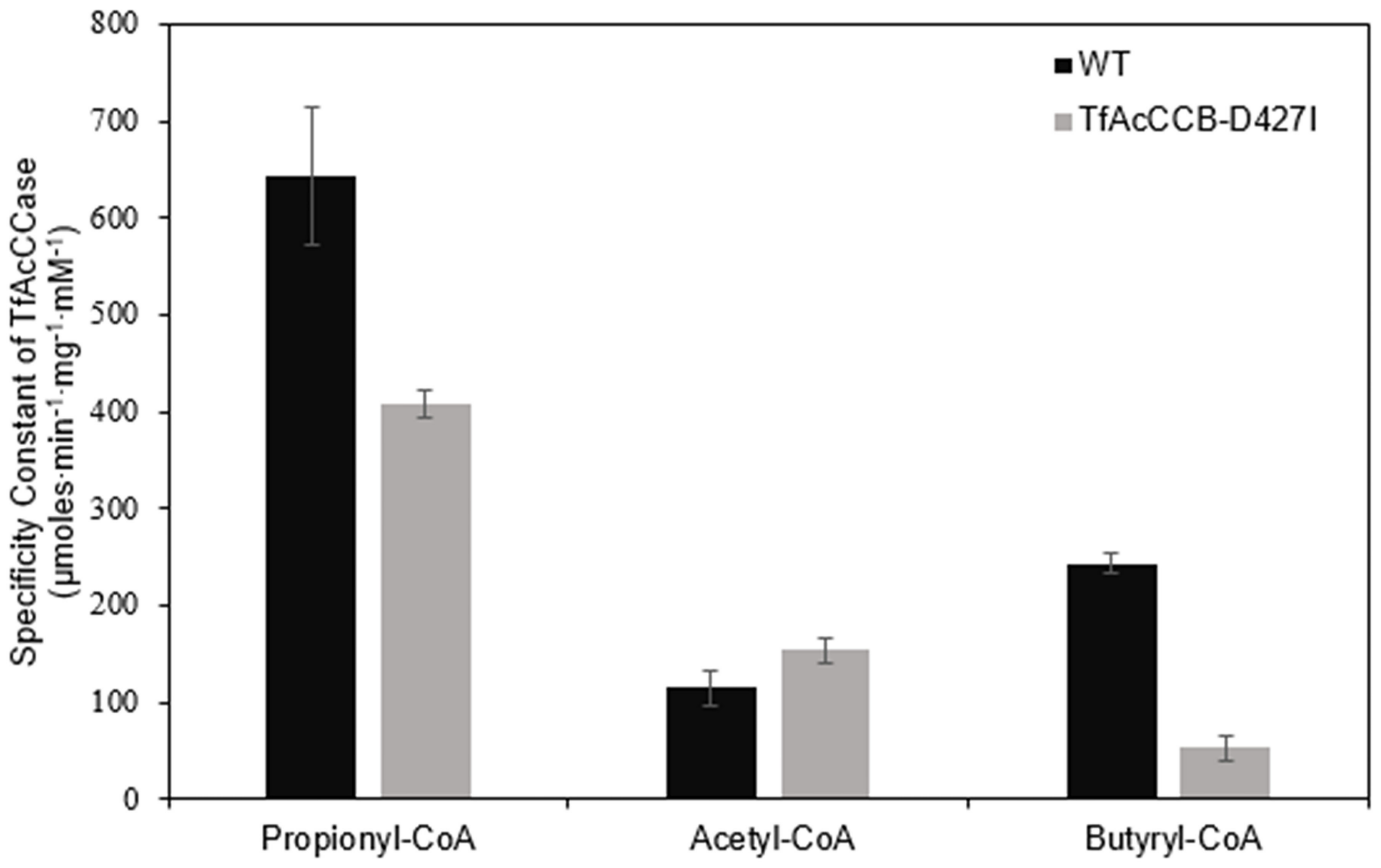
Comparison of the specificity constants of TfAcCCase preparations that are constituted with either the wild-type TfAcCCB or the TfAcCCB-D427I mutant subunit. Data-points are the average of triplicate assays (+SE) with either acetyl-CoA, propionyl-CoA or butyryl-CoA as the substrate. These experiments were conducted with two different enzyme preparations.

We evaluated if mutating the conserved D427 residue of TfAcCCB to Ile will affect the substrate specificity of TfAcCCase, as occurs with ScPCCase and ScAcCCase. Initial characterization of the purified TfAcCCase, assembled with the TfAcCCB-D427I mutant subunit established that the major form of the recovered holoenzyme has a molecular weight of 897 ± 39 kDa, which is indistinguishable from that of the wild-type enzyme (Figure 2B). This mutation therefore, did not affect the ability of the enzyme to assemble the normal quaternary organization. Moreover, this reconstituted TfAcCCase mutant enzyme demonstrates a relatively subtle change on substrate specificity. The TfAcCCB-D427I mutation increased the specificity constant (*V*_max_/*K*_m_) for the acetyl-CoA carboxylation reaction (by 150%) and decreased, by 40–80%, the specificity constant of the butyryl-CoA or propionyl-CoA carboxylation reactions. These alterations are primarily due to changes in *V*_max_, whereas the *K*_m_ values for each substrate were unaffected (Table 2, Figure 6).

**Table 2:** Michaelis-Menten kinetic constants for the carboxylation reaction catalyzed by TfAcCCase preparations that are constituted with either wild-type or TfAcCCB-D427I mutant subunit.

**Kinetic Constants**	**Acyl-CoA substrates**					
	**Propionyl-CoA**		**Acetyl-CoA**		**Butyryl-CoA**	
	**Wild-type**	**D427I**	**Wild-type**	**D427I**	**Wild-type**	**D427I**
*V*_max_ (μmoles.min^−1^.mg^−1^)	508 ± 23	298 ± 14	173 ± 12	247 ± 10	163.9 ± 2.6	50.8 ± 4.9
*K*_m_ (mM)	0.79 ± 0.08	0.73 ± 0.08	1.5 ± 0.2	1.58 ± 0.11	0.67 ± 0.02	0.95 ± 0.19
*V*_max_/*K*_m_	644 ± 71	408 ± 14	115 ± 17	154 ± 12	244 ± 10	53 ± 12

## Discussions

Two attributes contribute to the significance of enzymes in facilitating biological processes: (1) they enhance the rates of chemical conversions; and (2) they provide specificity to such chemical conversions. Selective pressure on existing diversity in both these attributes provides the mechanism to confer biological advantage that can facilitate Darwinian evolution in metabolic processes. Thus, selection based on chemical titers of a product can affect catalytic efficiency, and selection based on enzyme promiscuity vs. specificity can affect biochemical end-points of metabolism. The biotin-dependent family of enzymes offer exemplary illustrations of the pull and push that affects this latter attribute, i.e., the evolutionary advantage or disadvantage that confers specificity or promiscuity to a family of enzymes.

Biotin-dependent enzymes catalyze carboxylation, decarboxylation or transcarboxylation reactions (Cronan and Waldrop, 2002; Nikolau et al., 2003; Tong, 2013), and these enzymes are also classifiable on the basis of their organic substrates, and on the basis of the quaternary organization of the subunits into the holoenzyme complex. The majority of these enzymes catalyze the carboxylation of a specific substrate (i.e., acetyl-CoA carboxylase, propionyl-CoA carboxylase, 3-methylcrotonyl-CoA carboxylase, pyruvate carboxylase, and urea carboxylase (St. Maurice et al., 2007; Xiang and Tong, 2008; Fan et al., 2012; Waldrop et al., 2012; Tong, 2017). In contrast, the AcCCase enzyme that was the focus of the current study, displays a broader substrate specificity, being capable of carboxylating, with near equal efficiency, multiple acyl-CoA substrates (acetyl-CoA, propionyl-CoA, and butyryl-CoA). Additionally, there appears to be an evolutionary relationship between the quaternary organization of the enzyme-subunits, and the catalytic capability of each subunit (Tong, 2013). At one extreme are multi subunit carboxylases, typified by acetyl-CoA carboxylases from bacteria and plastids of some plants (Cronan and Waldrop, 2002; Nikolau et al., 2003), where four separate subunits share the BC, BCCP, and CT catalytic functionalities. At the other extreme are multi-functional carboxylases (e.g., acetyl-CoA carboxylases, urea carboxylase and some pyruvate carboxylases) that occur in the cytosol (St. Maurice et al., 2007; Xiang and Tong, 2008; Fan et al., 2012; Waldrop et al., 2012; Tong, 2017) and mitochondria of eukaryotic organisms (Hiltunen et al., 2009; Monteuuis et al., 2017). These enzymes harbor the BC, BCCP, and CT catalytic functionalities as domains on a single large subunit. Enzymes with intermediate quaternary organizations are typified by propionyl-CoA carboxylase (Huang et al., 2010; Gago et al., 2011), 3-methylcrotonyl-CoA carboxylase (Wurtele and Nikolau, 1990, 2000; Diez et al., 1994; Huang et al., 2012), and by bacterial pyruvate carboxylases (Choi et al., 2016). These enzymes have fused the BC and BCCP domains on one subunit, and the CT functionality is on a second subunit. Some of these latter enzymes, particularly from microbial organisms, also have a small, non-catalytic subunit.

The *T. fusca* YX AcCCase that was studied herein is exemplary of the latter, intermediate type enzyme, being composed of two catalytic subunits, AcCCA and AcCCB, and the non-catalytic AcCCE, and the reconstituted holoenzyme is promiscuous, capable of carboxylating acetyl-CoA, propionyl-CoA, and butyryl-CoA. These characteristics are similar to previously characterized AcCCases from Actinobacteria, such as *S. coelicolor* (Rodríguez and Gramajo, 1999; Rodríguez et al., 2001; Diacovich et al., 2002). Size exclusion chromatographic characterizations of co-expressed and co-purified TfAcCCase subunits indicate that the TfAcCCB subunit prefers to physically interact with the TfAcCCE subunit and predominantly assembles into a dodecameric TfAcCCB-TfAcCCE subcomplex (i.e., B_6_E_6_). In contrast, TfAcCCA subunit does not interact with the TfAcCCE subunit, and prefers to organize into an A_3_ complex, which can further coalesce to A_3_ dimers and trimers. These experiments imply that the TfAcCCE-TfAcCCB subcomplex assembles initially, and then the A_6_B_6_E_6_ holoenzyme complex is assembled.

Enzymatic assays of these recovered preparations reiterate the role of the non-catalytic TfAccE subunit in facilitating assembly of an active holoenzyme (Rodríguez et al., 2001; Gago et al., 2006; Gande et al., 2007). Thus, although the isolated TfAcCCA and TfAcCCB mixture can support catalysis, the addition of TfAcCCE into this mixture induces a 5- to 20-fold increase in the specificity constant (*V*_max_/*K*_m_) of the enzyme, depending on the acyl-CoA substrate. The increase in the specificity constant is directly proportional to the substrate preference of the holoenzyme; this being in decreasing order from propionyl-CoA to butyryl-CoA to acetyl-CoA. This role of a non-catalytic TfAcCCE subunit in facilitating holoenzyme assembly and activation of AcCCase catalysis is analogous to the recently characterized, non-catalytic BADC (biotin/lipoyl attachment domain containing) subunits, that facilitate similar assembly and activation of the plastidic heteromeric acetyl-CoA carboxylase (Shivaiah et al., 2020).

Prior characterizations of the *S. coelicolor* PCCase and AcCCase focused on identifying potential residues that may determine the different substrate specificities of these two enzymes and identified residues D422 and I420 of ScPccB and ScAcCCB, respectively, which are positioned in homologous positions of the two proteins (Diacovich et al., 2004). We identified the homologous residue in TfAcCCB as D427. Furthermore, the overall TfAcCCB sequence shares higher sequence similar with ScPccB (87%) than ScAcCCB (71%). Thus, although the structural features of TfAcCCB subunit is more like ScPccB, catalytically TfAcCCase behaves more like ScAcCCase, expressing more promiscuous substrate specificity, whereas ScPCCase is more specific for using propionyl-CoA as the substrate.

This apparent dilemma was experimentally explored, via site-directed mutational study, analogous to that reported earlier (Diacovich et al., 2004). Specifically, as compared to the wild-type enzyme, the D427I mutant TfAcCCB subunit did not affect the promiscuous nature of TfAcCCase, generating an enzyme that showed increased activity with acetyl-CoA (1.5-fold increase in *V*_max_/*K*_m_), accompanied with decreased activity with propionyl-CoA (40% decrease) and butyryl-CoA (80% decrease). This compares with the D422I mutation in ScPccB, which changes the ScPCCase enzyme from primarily carboxylating propionyl-CoA, to a more promiscuous enzyme, being capable of carboxylating acetyl-CoA, propionyl-CoA and butyryl-CoA (Diacovich et al., 2004). In contrast, the I420D mutation in ScAcCCB, narrows the substrate specificity of ScAcCCase so that it only carboxylates propionyl-CoA; the wild-type ScAcCCase is analogous to the TfAcCCase, being a promiscuous enzyme, carboxylating all three acyl-CoA substrates (i.e., acetyl-CoA, propionyl-CoA, and butyryl-CoA).

Integrally therefore, these findings indicate that the residue positioned at this site (a D or an I) is a determinant of substrate promiscuity, but the context of this residue (i.e., TfAcCCB, ScACCB or ScPccB) also has a role in determining this trait. This is consistent with molecular models, which presuppose that chemo-physical properties of multiple side-chain interactions are integrated to determine structure-function relationships (Kingsley and Lill, 2015), and thus enzyme substrate specificity vs. promiscuity (Tawfik, 2010).

## Data Availability Statement

The original contributions presented in the study are included in the article/Supplementary Materials, further inquiries can be directed to the corresponding author/s.

## Author Contributions

K-KS and BU carried out molecular cloning of different subunits and kinetic studies. K-KS contributed toward co-expression and co-purification of the holo-enzyme, site-directed mutagenesis, and size-exclusion chromatography. BJN perceived and supervised the project, and coordinated the writing. All authors contributed to the article and approved the submitted version.

## Conflict of Interest

The authors declare that the research was conducted in the absence of any commercial or financial relationships that could be construed as a potential conflict of interest.

## Abbreviations

ACCase: Acetyl-CoA Carboxylase
BC: Biotin Carboxylase
BCCP: Biotin Carboxyl Carrier Protein
CT: Carboxyl Transferase
AcCCB: Acyl-CoA Carboxylase
PccB: Propionyl-CoA Carboxylase.
