# Supplementary material for: Kinetic, Structural, and Mutational Analysis of Acyl-CoA Carboxylase From Thermobifida fusca YX
Source: Front Mol Biosci. 2021 Jan 12;7:615614. doi: 10.3389/fmolb.2020.615614 (PMC7835884; doi:10.3389/fmolb.2020.615614)
Supplement: Supplementary file 1 [file Table_1.DOCX]

*Supplementary materials*


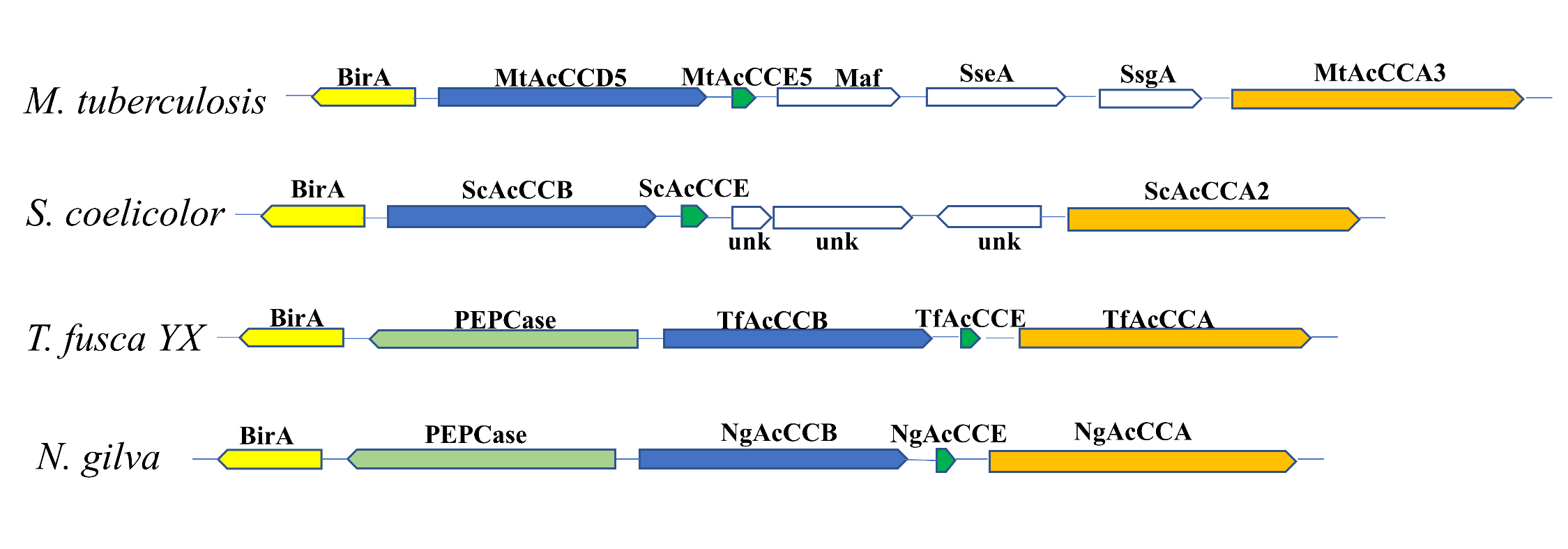


**Supplemental Figure S1.** Comparison of the genome organization of the AcCCase subunit genes in different bacteria.


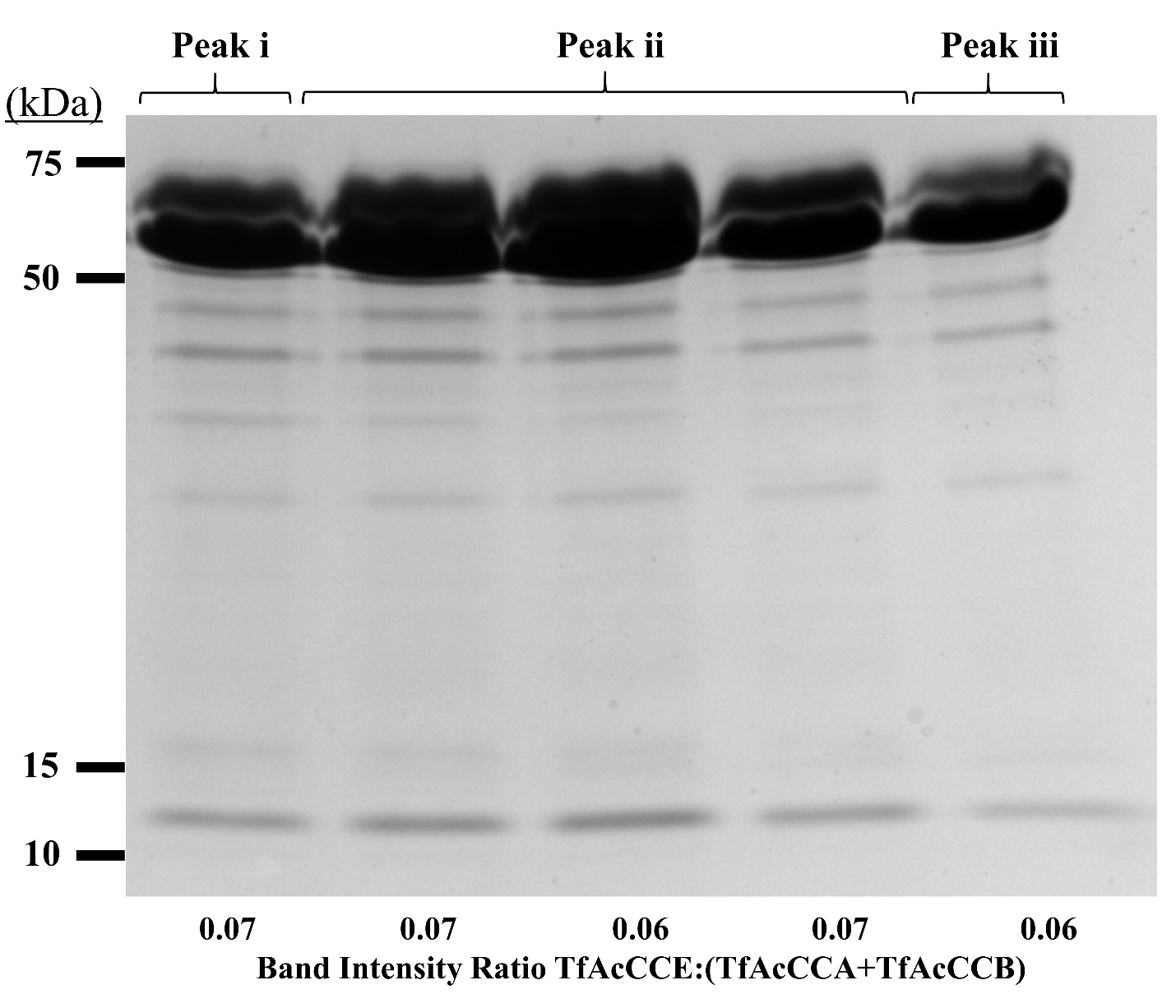


**Supplemental Figure S2.** Densitometric analysis of purified TfAcCCase. SDS-PAGE analysis of fractions eluted from gel filtration FPLC that encompassed Peaks i, ii, and iii of the reassembled wild-type TfAcCCase (Figure 2). The band intensity of the TfAcCCE subunit was normalized relative to the integrated TfAcCCA and TfAcCCB subunits bands.

**Supplementary Table S1.** Cloning vectors and primers information

| **Clone no** | **Description** | **Primers** |
| --- | --- | --- |
| 1 | pCDFDuet-1 vector with *AcCCE* ORF inserted in MCS1 to express N-terminal His-tagged fusion protein, and the *AcCCB* ORF was inserted in MCS2 and expressed as a C-terminal S-tagged protein | AcCCE FOR: 5`-ACCACAGCCAGGATCACACGAGCGACCACACCC-3`  AcCCE REV 5`-TTCTGTTCGACTTAATCACCCCGGATGAAATGAG-3` AcCCB FOR: 5`- AAGGAGATATACATAATGGCTACACAGGCGCCG -3` AcCCB REV: 5`- CTTTACCAGACTCGATCAGCGGAATATTACCGTG -3` |
| 2 | pCDFDuet-1 vector with *AcCCB* ORF inserted in MCS1 to express N-terminal His-tagged fusion protein, and *AcCCE* ORF was inserted in MCS2 and expressed as a C-terminal S-tagged fusion protein | AcCCB FOR: 5`-ACCACAGCCAGGATCACGCTACACAGGCGCCGG-3` AcCCB REV: 5`-TTCTGTTCGACTTAATTACAGCGGAATATTACCGT-3` AcCCE FOR: 5`- AAGGAGATATACATAATGACGAGCGACCACACC-3` AcCCE REV: 5`- CTTTACCAGACTCGATCCCCGGATGAAATGAGC -3` |
| 3 | pET30F vector with *AcCCA* ORF express with N-terminal His-tagged fusion protein under transcriptional control of the T7 promoter | AcCCA FOR: 5`-TCCAGGGCTCGGATCCAATGCGCAAAGTACTGATCG-3` AcCCA REV: 5`-GTGGTGGTGCTCGAGTCAACTTGATTTAATTTCGCAA-3` |
| 4 | pETDuet-1 vector with *AcCCA* ORF express without any tag | AcCCE FOR: 5`-AAGAAGGAGATATACATGCGCAAAGTACTGATCG-3`  AcCCE REV: 5`-AAGCATTATGCGGCCTCAACTTGATTTAATTTCGC-3` |
| 5 | pCDFDuet-1 vector with *AcCCB* ORF inserted in MCS1 to express N-terminal His-tagged fusion protein and *AcCCE* ORF inserted in MCS2 to express without any tag | AcCCB FOR: 5`-ACCACAGCCAGGATCACGCTACACAGGCGCCGG-3` AcCCB REV: 5`-TTCTGTTCGACTTAATTACAGCGGAATATTACCGT-3` AcCCE FOR: 5`-AAGGAGATATACATAATGACGAGCGACCACACC-3` AcCCE REV: 5`-CTTTACCAGACTCGATCACCCCGGATGAAATGAG-3` |
| 6 | pETDuet-1 vector with *AcCCE* ORF to express AcCCA without any tags | AcCCE FOR: 5’-AAGAAGGAGATATACATGACGAGCGACCACACCC-3’  AcCCE REV: 5’-AAGCATTATGCGGCCTCACCCCGGATGAAATGAGCG-3’ |
| 7 | Site directed mutagenesis primers used to make the D427I mutant of *AcCCB* ORF in clone number 5 | D427I FOR: 5`-AAGCGTTTGGCGGGGCTTATATTGTGATGGGTTCCAAG-3`  D427I REV: 5`-cttggaacccatcacaatataagccccgccaaacgctt-3` |

**Supplemental Table S2.** Michaelis-Menten kinetic parameters of AcCCase and PCCase enzymes from different bacteria

|  |  | **Substrate** | | | | | | | | |  |
| --- | --- | --- | --- | --- | --- | --- | --- | --- | --- | --- | --- |
|  |  | **Propionyl-CoA** | | | **Acetyl-CoA** | | | **Butyryl-CoA** | | |  |
| **Species** | **Enzyme** | **^a^V_max_** | **K_m_ (µM)** | **V_max_/K_m_** | **V_max_** | **K_m_ (µM)** | **V_max_/K_m_** | **V_max_** | **K_m_ (µM)** | **V_max_/K_m_** | **Citation** |
| *T. fusca* YX | AcCCase | 1051±47.8 | 797.8±81 | 1.32 | 356.9±25.7 | 1565±202 | 0.23 | 338.6±5.3 | 669.5±24.8 | 0.51 | This study |
| *S. coelicolor* A3(2) | AcCCase | 609±21 | 89.4±10.1 | 6.8 | 489±16.2 | 97.9±11.8 | 5 | 439.5±17.7 | 99.1±12.5 | 4.4 | *Diacovich et al.,* 2002 |
| *S. coelicolor* A3(2) | PCCase | 1063±21 | 76.5±5.5 | 13.9 | ND^a^ | ND | ND | 690.1±65.4 | 104.7±27.5 | 6.6 | *Diacovich et al.,* 2002 |
| *M. tuberculosis* | AcCCase | 680±43 | 240±35 | 2.8 | 120±8 | 220±55 | 0.5 | NA^b^ | NA | NA | *Gago et al.,* 2005 |
| ^a^Units of V_max_ are µmoles·min^-1^·mg^-1^ AcCCA subunit  ^b^ND: Not detectable  ^c^NA: Not applicable | | | | | | | | | | | |
